# Radiographic evaluation of enamel thickness of permanent teeth: relevance and applicability

**DOI:** 10.1590/2177-6709.29.3.e242422.oar

**Published:** 2024-07-08

**Authors:** Nicole Tonin IPLINSKY, Luiz Gonzaga GANDINI, Alyssa Schiavon GANDINI, Ana Thais BAGATINI, Pedro Henrique José de OLIVEIRA, Paula Cristina Henriques da SILVA, Ary SANTOS-PINTO

**Affiliations:** 1 São Paulo State University, Araraquara School of Dentistry, Graduate Program in Dental Science (Araraquara/SP, Brazil).; 2 São Paulo State University, Araraquara School of Dentistry, Department of Orthodontics (Araraquara/SP, Brazil).; 3 São Paulo State University, Araraquara School of Dentistry (Araraquara/SP, Brazil).

**Keywords:** Dental enamel, Radiography, Orthodontics, Enamel thickness, Interproximal enamel reduction, Esmalte dentário, Radiografia, Ortodontia, Espessura do esmalte, Redução interproximal do esmalte

## Abstract

**Objective:**

This descriptive observational study aimed to determine clinically relevant and applicable data of enamel thickness (ETH), considering the mesio-distal differences of anterior and posterior permanent teeth and their relationships.

**Material and Methods:**

The sample consisted of right-sided standardized radiographs of 34 individuals (21 females and 13 males), aged between 13 and 24 (average 16) years, with all permanent teeth intact and without crowding. Four periapical and four interproximal radiographs were obtained and digitized. ETH measurements (mesial to distal contact points at the dentin-enamel junction) were performed after correction for radiographic image magnification. The Students’ *t*-test was applied to the differences between paired means, with the Pearson correlation to evaluate the correlation between them.

**Results:**

The mesial and distal ETH increased from the anterior to the posterior teeth. Incisor ETH ranged between 0.60 and 0.84 mm. Canines, premolars, and molars were more than 1.0 mm thick, and molar enamel reached values between 1.26 and 1.44 mm.

**Conclusion:**

Distal ETH was significantly greater than the mesial ETH, and progressively thicker from the anterior to posterior teeth. Interproximal reduction (IPR) of the lower central and upper lateral incisors should be avoided, reduced, or performed on their distal surfaces. There is a positive and significant correlation between ETH and the mesial and distal surfaces of the teeth. Periapical radiographs and evaluation of the remaining ETH are necessary in cases of retreatment. The location and number of tooth size discrepancies should be considered in treatment planning and appropriately compensated with IPR.

## RESUMO

**Objetivo:**

Este estudo observacional descritivo teve como objetivo determinar dados clinicamente relevantes e aplicáveis da espessura do esmalte (EES), considerando as diferenças mésiodistais dos dentes anteriores e posteriores e suas relações.

**Material e Métodos:**

A amostra consistiu em radiografias periapicais padronizadas do lado direito de 34 indivíduos, 21 do sexo feminino e 13 do sexo masculino, com idade entre 13 e 24 anos (média = 16 anos), com todos os dentes permanentes íntegros e sem apinhamento. Quatro radiografias periapicais e quatro radiografias interproximais foram tiradas e digitalizadas. As medidas de EES (dos pontos de contato mesial e distal até a junção dentina-esmalte) foram realizadas após correção para adequar a ampliação da imagem radiográfica. Para as diferenças entre as médias pareadas, foi aplicado o teste *t* de Student com correlação de Pearson, para avaliar a correlação entre elas.

**Resultados:**

A EES mesial e distal aumenta dos dentes anteriores para os posteriores. A EES dos incisivos variou entre 0,6 e 0,84 mm. Caninos, pré-molares e molares apresentaram EES superior a 1,0 mm e a EES dos molares atingiu valores entre 1,26 e 1,44 mm.

**Conclusão:**

As EES distais são significativamente maiores que as mesiais e progressivamente mais espessas dos dentes anteriores para posteriores. A redução interproximal do esmalte (RIP) dos incisivos centrais inferiores e laterais superiores deve ser evitada, minimizada ou realizada em suas superfícies distais. Radiografias periapicais e avaliação da EES remanescente são necessárias nos casos de retratamento. A localização e o número de discrepâncias no tamanho dos dentes devem ser determinados e considerados no planejamento do tratamento, para que sejam adequadamente compensados durante o tratamento com RIP.

## INTRODUCTION

Removing the interproximal enamel from permanent teeth is a routine procedure in orthodontic clinics.^1^ ^-^ ^4^ This procedure is called stripping, and is indicated to correct dental crowding as well as to adjust tooth discrepancy, allowing for more favorable and stable overjet and overbite.^5^ ^-^ ^7^ The amount of interproximal enamel to be removed is strictly related to the amount of space needed to correct the existing dental crowding, the shape of the dental crown, and the thickness of the enamel.^8^ Effects such as dentinal exposure,^9^ ^,^ ^10^ temperature sensitivity, and reduced resistance to dental caries^1^ ^,^ ^2^ ^,^ ^8^ are adverse effects of excessive enamel removal. Existing clinical recommendations consider that no more than 50% of the enamel should be safely removed. Although there are studies indicating the thickness of tooth enamel that can be safely removed, there are no objective data on the amount of enamel and its variability (teeth with a thin or thick layer of enamel).

Changes reported in enamel thickness (ETH) between types of teeth and in different ethnic groups^4^ ^,^ ^11^ ^,^ ^12^ ^,^ ^14^ ^,^ ^15^ highlight the relevance of conducting this type of study for a specific population. To perform safe stripping, it is important to estimate the actual thickness of the enamel. Thus, orthodontists must follow precise parameters from scientific studies, and not based only on their sense of proportion.^4^ ^,^ ^11^ ^-^ ^13^ Radiographic images are the conventional method for evaluating dental hard tissues.^4^ ^,^ ^11^ ^,^ ^13^ ^,^ ^14^ ^,^ ^16^ ^,^ ^17^ Radiographs allow the quantification of ETH in a non-invasive way,^18^ and are recommended for samples involving living individuals. An advantage of this type of study is that it allows correlations to be established between groups of teeth from the same patient. 

Thus, the objective of the present study was to determine clinically relevant and applicable data on ETH in permanent teeth, considering the mesial-distal differences between anterior and posterior teeth and their relationships.

## MATERIAL AND METHODS

This prospective and descriptive observational clinical study was approved by the Research Ethics Committee of the Araraquara School of Dentistry (UNESP, Brazil) under number 05/08. The sample size was determined from data available in the literature, using 80% power for paired *t*-test. A design with a sample size of 34 can detect effect sizes of δ ≥ 0.5, with a probability of at least 0.81, assuming a two-sided criterion for detection that allows for a maximum Type I error rate of α = 0.05 for the paired *t*-test (jamovi 2.3 computer software 2022 retrieved from *https://www.jamovi.org*). The assessment of tooth ETH was performed on a sample of 34 individuals (21 females and 13 males), aged between 13 and 24 years (mean age of 16 years). All patients had complete permanent dentition up to second molars and no dental crowding, had not undergone any type of orthodontic treatment, and had no history of tooth stripping. The teeth should not present shape or structural anomalies or the presence of restorations, prosthetic restorations, or rotations that interfere with the visualization of the interproximal enamel. Individuals were recruited from dental students and individuals seeking orthodontic treatment at the Araraquara School of Dentistry.

Because previous studies did not show significant differences between right and left teeth ETH, and to reduce the subject exposition to radiation, right-sided radiographs were used as a standard.^11^ ^,^ ^12^ A systematic review of the literature^29^ ^,^ ^30^ showed a strong right-left symmetry for the same contralateral teeth and no sexual dimorphism in ETH. Data collection was performed using four periapical radiographs: one of the upper central and lateral incisors, and another of the lower central and lateral incisors; one of the upper canine and another of the lower canine; and four interproximal radiographs, one between the upper premolars and another between the lower premolars; one between the upper first and second molars, and another between the lower first and second molars. 

To ensure reliable measurements, radiographic film positioners (model Rinn XCP, Rinn Corp, Elgin, Illinois, USA) were used during the acquisition of the radiographs, to provide a standardized radiographic image with a low level of distortion. Magnification was controlled and corrected using as reference metallic spheres (with a standard diameter of 2 mm), placed before taking the radiographs in the central portion of the dental crowns, assisted by 1-mm-thick silicone molds made in the maxillary and mandibular study models. Radiographs were obtained using a Spectro 70 X-ray machine (Dabi Atlante, Ribeirão Preto, São Paulo, Brazil) at 70 kVp and 8 MA (radiographic films with F sensitivity were processed automatically, in a Dent-x 9000 processor) according to the manufacturer’s instructions. Then, the radiographs were digitized on a SnapScan 1236s flatbed scanner (Agfa, Gevaert N.V. Woburn, USA) at 2400 dpi. The images were imported into Adobe Photoshop software to be optimized and saved at 600 dpi.

ETH measurements (examiner blinded to upper and lower teeth and patient analyzed) were performed using Image Tool 3.0 software (University of Texas Health Science Center-San Antonio, Texas, USA) after appropriate correction for enlargement of the radiographic image with the same software (correction for magnification using the diameter of the spheres as reference). Measurements were taken at the contact points mesial and distal to the dentin-enamel junction, along the largest mesiodistal diameter of the dental crown, perpendicular to its long axis. Repeated measurements were performed randomly by the same examiner, with a two-week interval between them, to calculate the intra-examiner error. The reproducibility of the method was assessed using the Intraclass Correlation Coefficient (ICC) and *t*-test for repeated measurements (systematic error evaluation) for all measurements used in this study.

Paired *t*-tests were used to compare the mesial and distal ETH of each tooth and the corresponding surfaces of the upper and lower teeth. Descriptive statistics were obtained for the mesial and distal ETH of the upper and lower permanent teeth and for 50% of the values obtained. The correlation between the enamel thicknesses on the proximal surfaces of the teeth of the upper arch with the analogous teeth of the lower arch, as well as that of the anterior teeth with the posterior teeth of each arch, was evaluated using the Pearson’s correlation coefficient. The significance level for all tests was set at *p* ≤ 0.05.

## RESULTS

The reliability of the measurements was confirmed by the intraclass correlation coefficient (ICC, Cronbach alfa): overall ICC = 0.99 and ICC ≥ 0.90 for the lower teeth (except for distal of second premolar, ICC = 0.85) and ICC ≥ 0.90 for the upper teeth (except for distal of lower incisor and distal of second premolar, ICC= 0.87; and for mesial of second molar, ICC = 0.88). Kolmogorov-Smirnov statistics indicated that all measurements were normally distributed. Complementary *t*-tests for paired samples applied to replicate measurements were not significant, and the mean difference was ≤ 0.02 mm (SE = 0.01), indicating no systematic error in the measurements and excellent intra-examiner reliability. The overall coefficient of correlation of the replicate measurements was higher than 0.98 (*p*<0.05).

**Figure 1:**
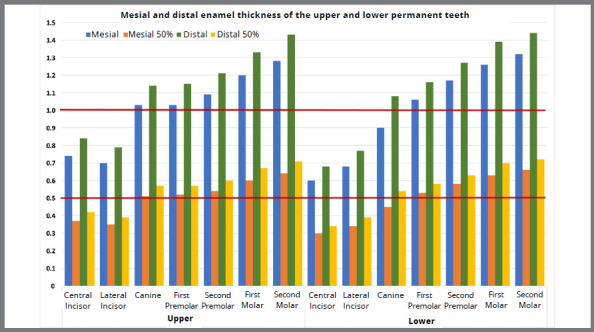
Total values and the corresponding to 50% of the enamel thickness on the mesial and distal surfaces of the upper and lower permanent teeth. Lower horizontal line indicates value limits up to 0.5 mm and upper horizontal line indicates value limits up to 1 mm.

The mesial and distal thickness of the enamel increased from the anterior to the posterior teeth in the upper and lower arches. The upper central incisors had a greater thickness of enamel than the lateral incisors. The ETH of the incisors ranged between 0.6 and 0.8 mm, and the thickness of the distal surface of the upper central incisor reached 0.84 mm. Canines, premolars and molars were thicker than 1 mm, except for the mesial portion of the lower canine, which was 0.9 mm. The thickness of the molar enamel varied between 1.26 and 1.44 mm. The thickness of the enamel on the distal surface was significantly higher than that on the mesial surface, but mainly less than 0.15 mm. Only on the upper canine had thickness equal to 0.18 mm (Table 1 and Fig 1).

**Table 1:** Mesial and distal enamel thickness of the upper and lower permanent teeth. Mesio-distal difference (t test) and correlation (Pearson)

	Proximal face									Mésio-distal		
	Mesial					Distal				Difference Correlationª			
Arch	Teeth	Mean	SD	Min	Max	Mean	SD	Min	Max	Mean	SE	r
Upper	IC	0.74	0.07	0.65	0.87	0.84	0.07	0.71	1.00	-0.10*	0.01	0.80*
	LI	0.70	0.07	0.59	0.83	0.79	0.07	0.66	0.95	-0.08*	0.01	0.86*
	C	1.03	0.10	0.84	1.20	1.14	0.11	0.97	1.48	-0.11*	0.01	0.90*
	IPM	1.03	0.08	0.86	1.23	1.15	0.08	0.95	1.29	-0.11*	0.01	0.45*
	2PM	1.09	0.09	0.88	1.29	1.21	0.07	1.10	1.38	-0.12*	0.01	0.80*
	1M	1.20	0.09	1.04	1.38	1.33	0.11	1.07	1.59	-0.13*	0.01	0.68*
	2M	1.28	0.08	1.08	1.46	1.43	0.10	1.17	1.61	-0.15*	0.01	0.74*
Lower	IC	0.60	0.06	0.51	0.70	0.68	0.06	0.57	0.79	-0.08*	0.01	0.82*
	IL	0.68	0.07	0.50	0.83	0.77	0.05	0.68	0.92	-0.09*	0.01	0.86*
	C	0.90	0.08	0.79	1.09	1.08	0.09	0.93	1.28	-0.18*	0.01	0.73*
	IPM	1.06	0.10	0.89	1.34	1.16	0.09	0.95	1.37	-0.10*	0.01	0.90*
	2PM	1.17	0.08	1.08	1.39	1.27	0.08	1.11	1.49	-0.10*	0.01	0.76*
	1M	1.26	0.11	1.05	1.43	1.39	0.11	1.13	1.53	-0.13*	0.01	0.90*
	2M	1.32	0.10	1.06	1.57	1.44	0.12	1.17	1.68	-0.12*	0.01	0.84*

Central Incisor(CI), Lateral incisor(LI), Canine(C), First premolar (1PM), Second premolar (2PM), First molar (1M) and Second molar (2M). Standard deviation (SD), Standard error (SE). ª Pearson’s Correlation; * p< 0.001.

Table 2 and Figure 1 present reference values with clinical relevance and applicability (values corresponding to 50% of the total ETH measured for permanent teeth). There was a positive and significant correlation between ETH and the mesial and distal thickness of the teeth (Table 1). Table 3 also indicates a positive and significant correlation between the mesial and distal enamel thickness of the lower molars and the mesial and distal thickness of all teeth (except the distal face of first lower molar and mesial face of upper central incisor and distal face of the upper canine). 

**Table 2:** Fifth percent values of mesial and distal thickness of the upper and lower permanent teeth.

		Mesial				Distal			
Arch	Teeth	Mean	SD	Min	Max	Mean	SD	Min	Max
Upper	CI	0.37	0.04	0.33	0.44	0.42	0.04	0.36	0.50
	LI	0.35	0.04	0.29	0.42	0.39	0.04	0.33	0.48
	C	0.51	0.05	0.42	0.60	0.57	0.06	0.48	0.74
	1PM	0.52	0.04	0.43	0.62	0.57	0.04	0.48	0.64
	2PM	0.54	0.05	0.44	0.65	0.60	0.04	0.55	0.69
	1M	0.60	0.04	0.52	0.69	0.67	0.05	0.53	0.79
	2M	0.64	0.04	0.54	0.73	0.71	0.05	0.58	0.81
Lower	CI	0.30	0.03	0.26	0.35	0.34	0.03	0.28	0.40
	LI	0.34	0.04	0.25	0.41	0.39	0.03	0.34	0.46
	C	0.45	0.04	0.39	0.55	0.54	0.05	0.46	0.64
	1PM	0.53	0.05	0.45	0.67	0.58	0.05	0.48	0.68
	2PM	0.58	0.04	0.54	0.70	0.63	0.04	0.55	0.75
	1M	0.63	0.05	0.52	0.72	0.70	0.06	0.56	0.77
	2M	0.66	0.05	0.53	0.79	0.72	0.06	0.58	0.84

Central Incisor (CI), Lateral incisor (LI), Canine (C), First premolar (1PM), Second premolar (2PM), First molar (1M) and Second molar (2M). SD = Standard deviation.

## DISCUSSION

From a clinical point of view, the anterior teeth are most frequently chosen to have their proximal surfaces reduced, either with stripping or interproximal reduction (IPR). This is done to correct crowding or lack of space in the anterior region or to correct anatomical changes or discrepancies of tooth size.^5^ ^-^ ^8^ The importance of the data in Table 1 is to show that IPR should be preferable on the distal faces of the tooth that are thicker than the mesial ones. The mesial surface of the lower canines was 0.09-mm thick, while the thickness for the upper canines was 0.10 mm. Hence, it is a good region for performing IPR if space is needed in the anterior region. In turn, IPR in the posterior region is less used by clinicians, because it is further away from the anterior region, despite data indicating a progressive increase in the thickness of the enamel from the anterior to the posterior teeth. Based on the results that posterior teeth are thicker than anterior teeth, these would be the teeth that could receive larger IPR. Canines, premolars and molars were more than 1-mm thick, except for the mesial of the lower canine (0.9 mm), and molars, which reached the highest values (1.26 to 1.44 mm).

In a systematic review and meta-analysis, Kailasam et al.^29^ found that the distal enamel was thicker than the mesial enamel by an average of 0.10 mm (from 0.09 to 0.12 mm). The smallest difference of 0.2 mm was observed for the upper and lower second molars, and a greater difference of 0.12 mm was observed in the upper central incisors and upper first premolars. The actual data point to similar results, indicating that distal ETH was significantly higher than mesial ETH, but mostly less than 0.15 mm, and only the upper canine was equal to 0.18 mm. The results also agree with those of Stroud et al.^4^ who reported a higher ETH of the first and second molars (0.3 and 0.4 mm, respectively) in relation to the anterior teeth in the lower arch. 

According to some authors,^1^ ^,^ ^23^ ^-^ ^25^ ^,^ ^29^ ^,^ ^30^ no more than 50% of the interproximal enamel can be safely removed. These values were calculated and displayed in Table 2. To obtain reference measurements to be used in determining the amount of enamel that can be safely removed, minimal individual variability in ETH values must be considered. Table 3 and Figure 1 show the variability in ETH and the reference values for IPR. With these values, teeth with the minimum ETH will maintain at least 50% of the original ETH after IPR. Considering 50% of the ETH (Table 2), IPR on the proximal surfaces of the lower incisors should be avoided or reduced. If necessary, IPR should be performed on their distal surface or in the lower lateral incisors. The safe amount of enamel reduction should be 0.3 mm for the mesial and distal surfaces of the upper and lower incisors. The only exception is the distal surface of the upper central incisors, which is thicker and for which up to 0.4 mm could be safely removed. Anterior teeth have thinner enamel than posterior teeth, and the lower central incisors have the thinnest enamel of all teeth. The upper and lower lateral incisors have similar ETH, whereas the upper central incisors have a higher thickness. For canines and premolars, the recommended amount of enamel removal would be 0.5 mm, except for the mesial region of the lower canine, which could reach 0.4 mm.

**Table 3:** Pearson’s correlation (r) between mesial and distal proximal faces of the lower and upper teeth.

	Lower	CI		LI		C		1PM		2PM		1M		2M	
Upper	Face	Mesial	Distal	Mesial	Distal	Mesial	Distal	Mesial	Distal	Mesial	Distal	Mesial	Distal	Mesial	Distal
CI	Mesial	0.31	0.20	0.31	0.17	0.38*	0.58**	0.36*	0.38*	**0.22**	0.20	0.36*	0.29	0.45**	0.36*
	Distal	0.23	0.21	0.19	0.20	0.43**	0.56**	0.48**	0.47**	0.41*	0.30	0.57**	0.46**	0.56**	0.55**
LI	Mesial	0.26	0.19	0.40*	0.36*	0.54**	0.61**	0.27	0.34*	0.26	0.19	0.45**	0.43**	0.47**	0.39**
	Distal	0.36*	0.30	0.40*	0.45**	0.73**	0.70**	0.47**	0.43*	0.46**	0.35*	0.49**	0.41*	0.57**	0.49**
C	Mesial	0.25	0.03	0.25	0.29	0.54**	0.44**	0.48**	0.37*	0.52**	0.28	0.49**	0.41*	0.57**	0.54**
	Distal	0.18	0.08	0.12	0.26	0.50**	0.27	0.29	0.17	0.46**	0.20	0.38*	0.27	0.45**	0.38*
1PM	Mesial	0.32	0.29	0.09	0.15	0.14	0.22	0.26	0.34	0.30	0.26	0.59**	0.51**	0.51**	0.44**
	Distal	0.22	0.40*	0.05	0.16	0.28	0.44**	0.30	0.30	0.27	0.25	0.54**	0.50**	0.53**	0.51**
2PM	Mesial	0.08	0.11	0.06	0.07	0.04	0.23	0.20	0.22	0.23	0.27	0.44**	0.46**	0.55**	0.48**
	Distal	0.12	0.12	0.12	0.10	0.15	0.40*	0.21	0.18	0.27	0.35*	0.48**	0.51**	0.61**	0.58**
1M	Mesial	-0.01	-0.07	-0.13	-0.12	0.08	0.14	0.00	0.05	0.19	0.16	0.59**	0.50**	0.54**	0.35*
	Distal	0.01	0.03	-0.14	0.02	0.15	0.11	0.04	0.13	0.26	0.28	0.65**	0.60**	0.64**	0.54**
2M	Mesial	-0.08	0.04	-0.05	-0.02	0.10	0.23	0.15	0.23	0.31	0.44**	0.63**	0.61**	0.53**	0.50**
	Distal	0.12	0.28	0.19	0.27	0.19	0.33	0.28	0.42*	0.38*	0.44**	0.65**	0.60**	0.73**	0.63**

Central Incisor (CI), Lateral incisor (LI), Canine (C), First premolar (1PM), Second premolar (2PM), First molar (1M) and Second molar (2M). * *p* < 0.05; ** *p* ≤ 0.01.

Harris and Hicks^14^ also observed larger ETH on the distal surface than on the mesial surface of the upper central and lateral incisors. These authors stated that on the distal surface of both incisors, the enamel is thicker, on average 1 mm, than on the mesial surface. This value was the same in the present study for the central incisor, but it was lower for the lateral incisor (0.08 mm). Considering the average values of the ETH of the anterior teeth, from the mesial of the canines from one side to the other, the reduction of the interproximal enamel would provide an additional space of 4.1 mm in the upper arch (SD 1.8mm) and 3.6 mm (SD 1.5mm) in the lower arch. From the mesial of the first molars from one side to the other, the reduction of the interproximal enamel would provide an additional space of 10.9 mm (SD 0.3mm) in the upper arch and 10.6 mm (SD 0.3mm) in the lower arch. Stroud et al^4^ reported that interproximal reduction of lower premolars and molars could result in a space gain of 9.8 mm, a value confirmed by the present study.

Professionals must be careful with IPR of tooth enamel, to avoid creating differences in the tooth size between the arches, especially when IPR is performed in a single arch. When reducing the lower teeth by a reasonable amount and not reducing the upper teeth, a Bolton discrepancy of the maxillary excess can be created. However, reduction of the proximal surfaces of the upper teeth is an important clinical option to be used by the orthodontist to treat size discrepancies between the upper and lower teeth, which is important to establish more favorable overjet and overbite relationships ^26^ ^-^ ^28^. Therefore, a Bolton discrepancy analysis should be performed before IPR, and planned IPR should be considered in the final assessment of Bolton discrepancy.^8^

According to Kailasan et al.,^29^ IPR is a routine procedure in clear aligner therapy, and the amount and location of this reduction are pre-planned by the operator. Reduction can be performed with the aim of promoting tooth movement, by creating the necessary space for this, and can also be performed to reduce the lack of space, correcting crowding. Thus, there is the possibility of choosing the areas where one wishes to gain space and the amount of enamel to remove. Dedicated planning software can virtually separate teeth and enlarge and rotate them for better visualization. Based on the present results and recommendations from systematic reviews,^29^ ^,^ ^30^ it would be clinically prudent to plan enamel reduction preferentially on the distal surfaces of the upper and lower teeth. In addition, diagnosis and treatment planning are important to determine the location and amount of tooth size discrepancies, so that they are adequately compensated during treatment.^26^ ^-^ ^28^

The positive and significant correlation between the ETH on the mesial and distal surfaces of the teeth, stronger in the upper arch than in the lower arch, indicates that there is a good probability that teeth with thin ETH in one region of the dental arch indicate that other teeth also have thin enamel (Tables 1 and 3). The clinician must also consider that differences in the mesial and distal thickness of the enamel can be found due to different wear patterns; therefore, younger patients should have thicker enamel, and adult patients should have thinner enamel.^4^ Schwartz^22^ suggested that ETH is related to occlusal function; thus, areas with greater occlusal forces would have thinner enamel and should be carefully examined.

Finally, caution must be taken with the increasingly frequent cases of retreatment, where the reduction of interproximal enamel may have been performed in a previous treatment. In these cases, is necessary to carefully analyze the remaining tooth enamel and how the planned IPR will impact on tooth discrepancy and the molar, canine, overjet and overbite relationship. A safe procedure in these cases of retreatment would be to obtain periapical radiographs and evaluate the remaining thickness of the enamel. With this piece of this information, the subsequent treatment should be planned. Studies on the topic are necessary because of the scarcity of data and its clinical relevance.

## RISK OF BIAS

Enamel thickness was not measured at the proximal contact point, which could result in bias when considering tooth shape (rectangular or triangular), for example in the presence of a dark triangular interdental space. Other aspects were not considered, as the location of the interdental bone crest, the periodontal characteristicsf the proximal gingival margin, and the three-dimensional aspect of the enamel.

## CONCLUSION

The distal ETH is significantly larger than the mesial, and progressively thicker from the anterior to posterior teeth. There is a positive and significant correlation between ETH on the mesial and distal surfaces of the teeth; therefore, teeth with thin ETH in one region of the dental arch indicate that the other teeth also have thin enamel. Based on the present results and on recommendations from systematic reviews, it would be clinically prudent to plan enamel reduction preferentially on the distal surfaces of the upper and lower teeth. IPR on the proximal surfaces of the lower central and upper lateral incisors should be avoided or reduced. When necessary, IPR should be performed on their distal surfaces.

Diagnosis and treatment planning are important for determining the location and amount of tooth size discrepancies, so that they are adequately compensated during treatment. A safe procedure in cases of retreatment would be to obtain periapical radiographs and evaluate the remaining thickness of the enamel, which should be considered in planning the subsequent treatment.
